# Loss of ‘Small-World’ Networks in Alzheimer's Disease: Graph Analysis of fMRI Resting-State Functional Connectivity

**DOI:** 10.1371/journal.pone.0013788

**Published:** 2010-11-01

**Authors:** Ernesto J. Sanz-Arigita, Menno M. Schoonheim, Jessica S. Damoiseaux, Serge A. R. B. Rombouts, Erik Maris, Frederik Barkhof, Philip Scheltens, Cornelis J. Stam

**Affiliations:** 1 Departments of Radiology, VU University Medical Center, Amsterdam, The Netherlands; 2 Department of Radiology, CITA-Alzheimer Foundation, San Sebastian, Spain; 3 Stanford Cognitive and Systems Neuroscience Laboratory, Stanford University, Palo Alto, California, United States of America; 4 Leiden Institute for Brain and Cognition (LIBC), Department of Radiology, Leiden University, Medical Center, Leiden University-Institute for Psychological Research, Leiden, The Netherlands; 5 Donders Institute for Brain, Cognition and Behavior, Radboud University, Nijmegen, The Netherlands; 6 Neurology, VU University Medical Center, Amsterdam, The Netherlands; 7 Clinical Neurophysiology, VU University Medical Center, Amsterdam, The Netherlands; University of Washington, United States of America

## Abstract

**Background:**

Local network connectivity disruptions in Alzheimer's disease patients have been found using graph analysis in BOLD fMRI. Other studies using MEG and cortical thickness measures, however, show more global long distance connectivity changes, both in functional and structural imaging data. The form and role of functional connectivity changes thus remains ambiguous. The current study shows more conclusive data on connectivity changes in early AD using graph analysis on resting-state condition fMRI data.

**Methodology/Principal Findings:**

18 mild AD patients and 21 healthy age-matched control subjects without memory complaints were investigated in resting-state condition with MRI at 1.5 Tesla. Functional coupling between brain regions was calculated on the basis of pair-wise synchronizations between regional time-series. Local (cluster coefficient) and global (path length) network measures were quantitatively defined. Compared to controls, the characteristic path length of AD functional networks is closer to the theoretical values of random networks, while no significant differences were found in cluster coefficient. The whole-brain average synchronization does not differ between Alzheimer and healthy control groups. Post-hoc analysis of the regional synchronization reveals increased AD synchronization involving the frontal cortices and generalized decreases located at the parietal and occipital regions. This effectively translates in a global reduction of functional long-distance links between frontal and caudal brain regions.

**Conclusions/Significance:**

We present evidence of AD-induced changes in global brain functional connectivity specifically affecting long-distance connectivity. This finding is highly relevant for it supports the anterior-posterior disconnection theory and its role in AD. Our results can be interpreted as reflecting the randomization of the brain functional networks in AD, further suggesting a loss of global information integration in disease.

## Introduction

Alzheimer disease (AD) has been characterized as a disconnection syndrome ([1] for AD; [2] for age-related cognitive decline), implying network-wide functional changes due to local structural changes. In AD, both selective decreases and increases in brain activity have been reported in a variety of behavioral conditions. In the absence of goal-directed behavior (i.e. resting-state condition), two different brain networks, the default mode network (DMN) [3], [4] and the dorsal visuo-spatial attention system [5], [6], show decreased activity in AD patients compared to age-matched healthy subjects ([7], [8] but see also [9]). Hypoactivity on both the DMN and visuo-spatial attention system has been associated with a decrease in spontaneous thoughts [10] and cognitive decline [11], [12], [13]. Relative activity increases in a variety of brain regions (dorso-lateral prefrontal cortex, anterior cingulate cortex and lingual gyrus) have been interpreted as the engagement of compensatory and cognitive reserve mechanisms [14], [15], [16]. Therefore, the consequences of AD are not only those directly related to the loss of functional relationships between previously connected brain regions, but also involve abnormal or compensatory reorganizations of non-compromised functional connectivity networks.

A small number of studies have pioneered the exploration of the principles and dynamics of global network changes in AD. In a very recent report applying graph analysis (a whole-brain network analysis technique; see below), Supekar et al. describe disruptions in local network connectivity in AD but no change in the connectedness of the global brain network [17]. These results are in contrast with those obtained by Stam et al. [18] and He et al. [19], both indicating long-distance connectivity differences. We examine this controversy in the context of the disconnection syndrome hypothesis by characterizing both local and global connectivity changes of the functional network in AD using graph analysis on blood-oxygen level dependent (BOLD) functional magnetic resonance imaging (fMRI).

### Graph Analysis

Graph analysis enables the study of complex systems described by way of pairwise relations between discrete elements. It can be used to explore and quantify the structure of whole-brain patterns of anatomical and functional connectivity [20], [21]. To express functional connectivity between discrete brain areas we calculate the synchronization likelihood (SL) [22]. SL is a measure of linear and non-linear dynamic dependencies between fMRI BOLD time-series acquired during resting-state condition. By using graph analysis, the characteristics of the whole-brain synchronization patterns are then modeled into a network composed by ‘vertices’ or brain regions responsible for an activity pattern connected by ‘edges’ representing inter-vertex synchronization levels.

### Small-world Networks

In their seminal paper, Watts and Strogatz used graph analysis to describe the emergent properties of a certain network type, i.e. increased signal-propagation speed, processing power and syncronizability as a small-world phenomenon. The authors characterized this network type as ‘small-world’ based on two measures, typical inter-vertex separation (path length, L) and the degree of grouping between network vertices (clustering coefficient, C) [23]. Compared to ordered and random networks, small-world networks are characterized by highly clustered vertex assemblies (high C) with a limited number of global shortcuts between clusters (relatively low L), which favors functional synchronization. Small-world network structures have been described in healthy anatomical [24], [25] and functional brain networks [26], [27], [28], [29] and its organizational principles of local specialization and global integration [30] are recognized as the bases supporting higher cognitive functions [31]. Therefore, the application of graph theory on functional connectivity fMRI data set allows us to classify the clustering and connectedness of the whole-brain functional network, and to judge its emergent functional properties. The subdivision of the brain activity patterns in discrete functional networks, like the DMN or the dorsal visuo-spatial attention system, excludes the analysis of their possible interactions and the emergent global configuration [32]. By contrast, examination of the brain-wide consequences to local network dysfunction can shed light on aspects of AD beyond the primary function loss, including compensatory mechanisms, early detection and prognosis.

There is increasing evidence that the altered brain function in diseases as Schizophrenia, AD or attention deficit hyperactivity disorder is characterized by the conversion from small-world network architecture to less optimal functional topologies [17], [18], [33], [34], [35], [36]. With the present study we intent to determine the functional characteristics of the whole-brain networks by applying graph analysis to fMRI data acquired in resting-state condition. Furthermore, we investigate whether local dysfunction and functional disconnection in AD brain synchronization induce a randomization of the original small-world network structure.

## Materials and Methods

### Ethics Statement

The study was approved by the Medical Ethics Review Committee of the VU University Medical Centre, Amsterdam. All participants in this study were required to provide a written informed consent in accordance with the VU university Medical Center Medical Ethical Committee; patients under supervision of a lawful caregiver if necessary.

### Subjects

This study was performed on the data collected from a subpopulation of subjects of a previously published study [7], [11], [32].

The selected data set is composed of two groups of right-handed participants: 21 older healthy subjects without memory complaints (age 70.7±6.0, range 60 to 81 years; 13 female) and 18 patients with mild AD (age 70.7±7.2 years, range 59 to 79 years; 9 female). Mini-Mental State Examination (MMSE) [37] scores are significantly different in AD patients compared to control subjects (AD 22.6±3.2, range 17–29; controls 28.7±1.4, range 25–30; p<0.001). Details regarding recruitment, diagnostic criteria and analysis of comorbid lesions (i.e. presence of vascular lesions) have been previously reported ([7], [11]; see next sections for details).

All subjects underwent an MRI session of approximately 35 minutes total. Subjects' respiration and pulse rate data were registered by means of respiration belt and pulse sensor. For the resting-state scan, lasting about 10 minutes, subjects were instructed to lie still with their eyes closed, not to think of any one thing in particular and not to fall asleep. Upon completion of the fMRI procedure none of the participants reported falling sleep during the scan acquisition.

### Neuropsychological assessment

All participants underwent a Mini Mental Status Examination (MMSE), a Geriatric Depression Scale (GDS), the Dutch version of the New Adult Reading test (NLV - premorbid IQ test) and a neuropsychological test battery including tests measuring attention/concentration, processing speed, episodic memory, executive functioning and praxis [7], [11], [32].

### Imaging methods

Imaging was performed on a 1.5T Sonata (Siemens, Erlangen, Germany) scanner. For the functional scan, T2*-weighted echo planar images (EPI) were acquired with the following sequence parameters: TR = 2850 ms; TE = 60 ms; flip angle = 90°; 36 axial slices; voxel size 3.3 mm isotropic – 200 volumes were acquired in 9 minutes and 30 seconds. Additionally a high-resolution T2*-weighted EPI and a high-resolution T1-weighted magnetization prepared rapid acquisition gradient echo (MPRAGE) image were acquired. The sequence parameters of the high-resolution EPI were: TR = 7230 ms; TE = 45 ms; flip angle = 90°; 64 axial slices; voxel size 1.6×1.6×2.2 mm. The sequence parameters of the T1 weighted image were: TR = 2700 ms; TE = 3.97 ms; flip angle = 8°, 160 coronal slices; voxel size 1×1.5×1 mm.

### Pre-processing of fMRI sequences and time-series extraction

Patients were recruited at the Alzheimer Center of the VU University Medical Center, Amsterdam, Netherlands. Diagnostic criteria for AD were that of NINCDS-ADRDA [38], with MMSE scores >18 and CDR <2. Healthy subjects were recruited by two means: (1) asking family members of patients and (2) advertisements posted in the medical center, the medical faculty of the university and activity centers for the elderly in the community. Participants were excluded if they had any significant medical, neurological (except for the diseases under study here in the patient groups) or psychiatric illness; a history of brain damage; or if they were taking medication known to influence cerebral function (except for AD medication in the AD group). T2-weighted fluid attenuation inversion recovery (FLAIR) scans of each subject were reviewed by a neuroradiologist (FB) to assess the presence of vascular lesions. Across all groups some, probably age-related, WM abnormalities were observed (29 subjects with Fazekas-score range 1-3 (1.21±0.41); and 3 subjects with 1–3 lacunes) [39]. T1-weighted MPRAGE scans were also inspected by a radiologist for structural abnormalities (FB). None of the scans included in this experiment presented outspoken posterior cingulate-precuneus or parietal patterns of atrophy.

The image pre-processing was carried out similarly as in the previously published resting-state studies [32], using tools from FMRIB's Software Library (FSL version 3.2) [40]. The following pre-statistics processing was applied: motion correction [41]; removal of non-brain structures [42]; mean-based intensity normalization of all volumes by the same factor (i.e. 4D grand-mean scaling in order to ensure comparability between data sets at the group level); high-pass temporal filtering (Gaussian-weighted least-squares straight line fitting, with sigma = 75.0s); and prewhitening to remove temporal autocorrelations.

For each participant the mean (across voxels) voxel absolute (each time point with respect to the reference image) and relative (each time point with respect to the previous time point) was calculated by MCFLIRT (motion correction software; FSL). Displacements across participants were found to be small (mean ± S.D. of displacement AD (absolute) = 0.43±0.28 mm; AD (relative) = 0.11±0.07 mm; OC (absolute) = 0.33±0.16 mm; OC (relative) = 0.11±0.07 mm); no significant differences were found between the experimental groups (2-tailed t-test; absolute displacement p = 0.21; relative displacement p = 0.82).

No global spatial smoothing was applied in order to avoid spurious synchronization between neighboring voxels.

Following the pre-processing the functional scan was first aligned to the high resolution EPI scan, then to the high resolution T1-weighted image, which was subsequently registered to the MNI152 standard space (average T1 brain image constructed from 152 normal subjects at Montreal Neurological Institute) using affine linear registration as implemented in FLIRT [41]. To minimize the effect of disease-related atrophy, we performed an additional, independent registration procedure thereby transforming the functional data set to a custom brain template or ‘midspace’. By calculating the geometric mean of the previously computed affine transformation matrices to the MNI152 standard space, midspace was defined as the transformation that approximates the average size and shape of the individual subjects' spaces. Hence the registration of functional data sets to midspace template accounts for the differential atrophy and brain shape between subjects by reducing the differences between the individual spatial transformations.

After registration, non-overlapping regions of interest (ROI) based on the anatomical automatic labeled brain (AAL; http://www.cyceron.fr/freeware/) [43] were extracted per subject. To calculate the coupling of brain activity between the regions defined, single time-series per subject and ROI were calculated by averaging all voxels' time-series within the ROIs. In total, 116 fMRi time-series (54 per hemisphere and 8 midline structures) consisting of 200 time-points were available per subject. In view of possible concerns over the accuracy of the result of the affine linear registration on the cerebellum, a second set of ROIs per subject excluding the cerebellar regions (n = 26) were calculated. In this second set of averaged voxels' time-series, the total number or ROIs defined per brain was reduced to 90.

### Functional connectivity computation: SL

Linear and non-linear dynamic correlations between all pair wise combinations of time-series were measured with the SL [22], [44] (for mathematical details on the SL computation, see File S1). Briefly, the SL calculation involves dividing each time-series into a series of patterns (short segments of the time-series containing a few cycles of the dominant frequency) and searching for the recurrence of these patterns. The SL is then the average probability that pattern recurrence in time-series X coincides in time with pattern recurrence in time-series Y.

**Figure 1:**
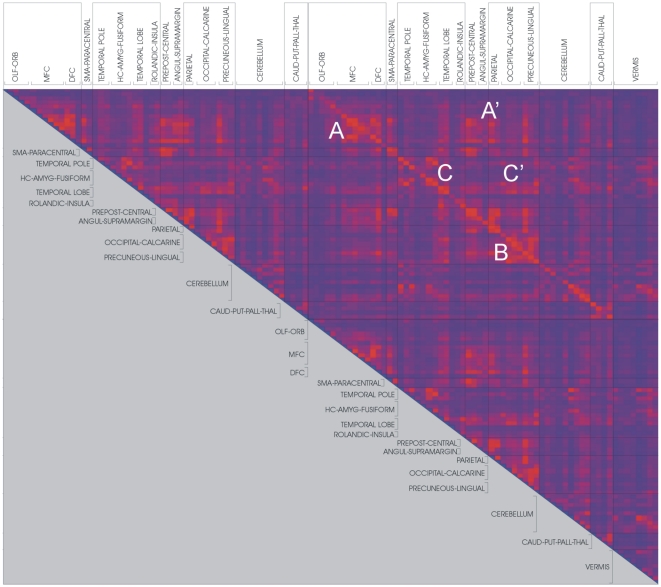
Synchronization matrix of AD group. Intensities of SL values between any given pair of brain regions are color-coded (blue, SL = 0; red, SL = 1). Labels and guidelines represent groups of AAL brain ROIs. Upper left corner represents the synchronization level between brain regions in the right hemisphere; lower right corner is the synchronization within the left hemisphere; upper right corner (diagonal line) corresponds to the synchronization between hemispheres. On this region, main synchronization clusters are indicated: A) frontal cortex; A’) frontal cortex with pre and post-central gyri and parietal cortex; B) parietal and occipital cortices and precuneus; C) temporal lobe; C’) temporal lobe with parietal and occipital lobes. The overall synchronization pattern reveals a relative increase of connectivity in the frontal cortices in AD (A); compared to the control group (Figure 2), decreases of connectivity in AD are spread throughout temporal cortices (C and C’) and particularly the parietal and occipital region (B). Note that the synchronization matrices are symmetrical – lower half of the matrices are grayed out for convenience.

**Figure 2:**
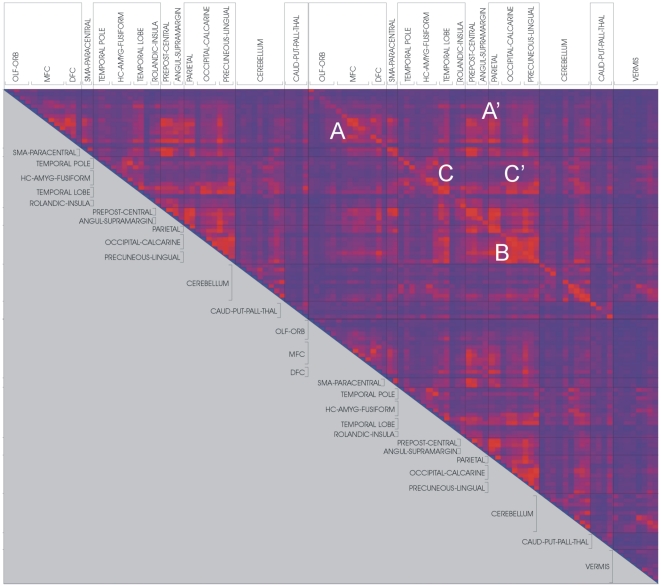
Synchronization matrix of control group. See legend of Figure 1.

The synchronization likelihood was computed with DIGEEGXP2 software (CS). Values of the SL range between Pref when there is no coupling and 1 in the case of fully synchronized time-series. Therefore, Pref is the value of the small but nonzero likelihood of coincident pattern recurrence between independent time-series. In the present study SL measures were calculated at Pref 0.01 to correct for low SL values that correspond to spurious synchronization. For the state space embedding (reconstruction of all possible values of a system) we used a time lag of 1 sample and an embedding dimension of 6; we further applied the Theiler correction for autocorrelation (w = 6). SL values were computed for both groups of control subjects and AD patients, resulting in two synchronization matrices where each entry contains the group average value of the SL for two given time-series (Figures 1 and 2).

### Graph analysis computation: network descriptors C and L

The SL values express the degree of functional connectivity between the activity patterns of any two anatomically independent brain regions. To represent the global connectivity, we used graph theory analysis to convert the synchronization matrices into binary graphs or networks consisting of ‘vertices’ (elements) and ‘edges’ (undirected link between two vertices).

**Figure 3:**
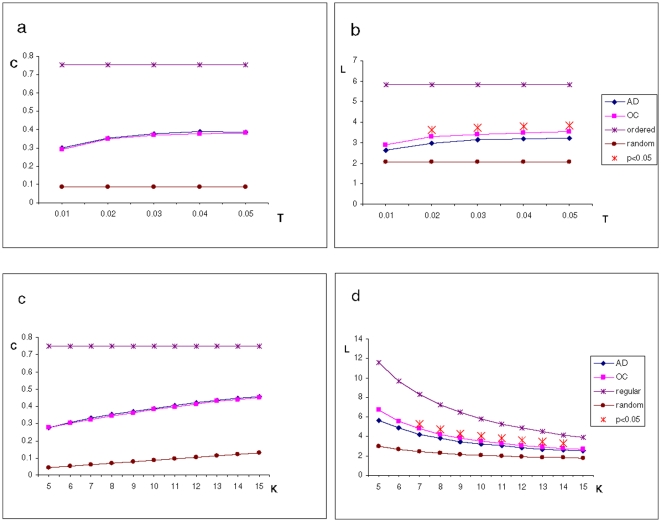
Cluster coefficient and path length. Subplots (a) and (b): Mean cluster coefficient and path length calculated for a range of synchronization threshold values (0.05<T<0.01; K constant  = 10). Subplots (c) and (d): cluster coefficient and path length computed for a range of K values (5<K<15; T constant  = 0.05). Path length L is significantly shorter in AD within a wide range of T and K values, therefore closer to the characteristics values for random networks. Cluster coefficient C is equal for both groups.

Three different sets of binary graphs/networks were created per subject, representing the whole brain synchronization patterns, the influence of the differential brain atrophy between AD and control groups (registrations to MNI152 standard space or midspace) and the contribution of the cerebellum (with or without cerebellar regions): (1) 116 ROIs in standard space, (2) 90 ROIs in standard space, and (3) 90 ROIs in midspace. In these idealized networks, the vertices correspond to the ROI’s time-series. The synchronization matrices are converted to a graph by considering a threshold T. If the SL between a pair of ROIs exceeds T an edge is said to exist between their representing vertices; otherwise no edge exists between them. Because there is no unique way to choose T, we examined the possible networks configurations by constructing graphs for a range of values of T (0.01<T<0.05 with increments of 0.01; Figure 3a,b) within which we explored the consistency of the network characteristics.

The topologies of the resulting connectivity networks were characterized in terms of their corresponding clustering coefficient (C) and characteristic path length (L). The clustering coefficient measures the degree of local interconnectedness of a vertex by analyzing the properties of its “neighbor” vertices (vertices joined to it by 1 edge). Clustering coefficients per vertex are calculated as the ratio of the number of existing edges between its neighbor vertices and the maximum possible number such an edges. The global clustering coefficient of the networks was computed by averaging the C of all vertices of the graph. The characteristic path length of a network represents its global connectivity/integration, including the influence of both local and long-range functional connections. L corresponds to the harmonic mean of the inverse shortest distance (in number of edges) for each vertex pair, assigning a value of zero when two vertices are not connected [45], [46].

The SL values can be different between subjects; this would mean that at a given value of T, a graph could have more or less edges than other, thereby influencing the differences in C and L. To guarantee the comparability between both groups, the graph parameters C and L were also computed as a function of the average number of edges per vertex (K). The networks tested must comply with a configuration such that N>K>ln(N)>1, where K>ln(N) guarantees that a random graph will be connected. This configuration corresponds to sparsely connected networks composed by many vertices [23]. We have tested a AAL template-based brain network for a range of 5<K<15 with increments of 1. Therefore, graphs of both AD and control groups *share a fixed number of edges*, despite possible differences in the mean SL values, ensuring that the differences in C and L between the groups solely reflect differences in graph topology (Figure 3c,d).

For comparison, 50 random graphs were computed based on the original synchronization matrices for each subject. These random networks preserved the number of vertices and edges as well as the degree distribution of the original networks [47]. Mean C and L for AD and control groups were recalculated as a ratio of the original values and their randomized surrogates C-s and L-s (corrected cluster coefficient γ = C/C-s; corrected path length λ = L/L-s ). The values obtained of C and L as a function of degree K were compared with the theoretical values of C and L for ordered (C = 3/4, L = N/2K) and random (C = K/N, L = ln(N)/ln(K)) graphs.

### Analysis of regional differences in synchronization

Based on the group differences demonstrated with graph analysis, a post-hoc analysis was performed at the level of regional synchronization. The distribution of SL differences between AD and control groups was tested using 2-tailed t-tests. The same analysis was repeated for the all the available data sets: 116 ROIs in standard space, 90 ROIs in standard space, and 90 ROIs in midspace. Despite the differences in number of functional ROI couples tested and correction of SL values due to the spatial registration, all tests delivered a similar pattern of SL regional differences between AD and controls at p<0.05 uncorrected.

We further compared AD patients and controls on the clustering coefficient C and characteristic path length L. We controlled for type I rate inflation due to the multiple comparisons at the different thresholds T (for the SL) and K (for the average number of edges per vertex). We used a modified version of the cluster-based permutation test proposed by Maris et al. [48]. The basic idea of this method is to reduce the multiple comparison problem to a single comparison problem by making use of a single test statistic that depends on the differences between the groups at all levels of T or K. The significance of this single test statistic is then evaluated under the permutation distribution. Our test statistic starts from calculating two-sample t-statistics separately for each of the levels of T or K. Subsequently, these t-statistics are threshold at some prior value (in our case, the 2.5-th and the 97.5-th quantiles of their corresponding sampling distributions). The supra-threshold values are then clustered on the basis of adjacency (i.e., supra-threshold values for adjacent values of T or K are joined in one cluster). Next, cluster-level statistics are calculated by summing the values within every cluster. Finally, the single test statistic combining information over all levels of T or K is obtained by taking the maximum of the positive cluster-level statistics (for positive one-sided and two-sided null hypotheses) and/or the minimum of the negative cluster-level statistics (for negative one-sided and two-sided null hypotheses). This test statistic is then evaluated under the permutation distribution that is obtained by randomly repartitioning AD patients and controls over two groups. By modeling data sets representing all possible combinations of patients and controls, we not only test for the robustness of the graph parameters differences, but also the likeliness of the original synchronization differences in our population.

## Results

### Synchronization patterns

The mean functional coupling between the different brain regions is summarized in Figures 1 and 2 (all figures provided correspond to the graph analysis derived from the global synchronization patterns of the set of 116 ROIs registered to standard space - including cerebellar ROIs). The synchronization matrices of AD (Figure 1) and control groups (Figure 2) express the intensity of the synchronization in color coded SL values (blue, SL = 0; red, SL = 1). The upper left corner of the matrices represents the synchronization level between brain regions in the right hemisphere, while the left hemisphere internal synchronicity is represented in the lower right corner. The upper right corner of the matrices, crossed by a diagonal line, corresponds to the synchronization between left and right hemispheres. Within these functional domains, three major clusters representing intense functional coupling between defined anatomical regions are defined: A) corresponds to the coupling within different regions of the frontal cortex, while A') represents relationships between frontal cortex with pre and post-central gyri and parietal cortex. The wider synchronization cluster visible, B) comprises the anatomically wide functional relationship between parietal and occipital cortices, including the precuneus. Two important minor clusters C) and C') represent the internal connectivity within the temporal lobe and its functional relationship with the parietal and occipital lobes respectively.

### Global synchronization mean

The global average synchronization, S, does not differ between Alzheimer and healthy control groups (Table 1; AD group S = 0.09±0.01; control group S = 0.09±0.02). The global synchronization levels remain unaltered depending on the registration to standard or midspace or the inclusion of cerebellar regions (standard space, 90 ROIs: AD group S = 0.10±0.02; control group S = 0.10±0.02; midspace, 90 ROIs: AD group S = 0.09±0.02; control group S = 0.09±0.02)

**Table 1:** Average synchronization, cluster coefficient and path lengths.

			AD			OC		p values
	Reg_#ROIs	mean		sd	mean		sd	
**S**	Ss_116	0,09		0,01	0,09		0,02	0,82
	Ss_90	0,10	±	0,02	0,10	±	0,02	0,82
	Ms_90	0,09		0,02	0,09		0,02	0,83
**γ**	Ss_116	4,44		0,50	4,38		0,50	0,69
	Ss_90	3,63	±	0,47	3,64	±	0,38	0,98
	Ms_90	3,72		0,39	3,57		0,30	0,19
**Λ**	Ss_116	1,53		0,15	1,69		0,22	0,01*
	Ss_90	1,43	±	0,17	1,61	±	0,25	0,01*
	Ms_90	1,44		0,14	1,61		0,27	0,02*

Data are presented as mean ± sd. AD, Alzheimer patient; OC, age-matched control; t-test (2-tailed;

*, p<0.05 between groups difference). While maintaining similar brain average synchronization levels (S), the Alzheimer group consistently shows a significantly shorter corrected path length (λ) that controls and similar cluster coefficient (γ) regardless of the registration procedure (Ss – standard space; Ms – midspace) and the inclusion of cerebellar regions (#ROIs 116 – including cerebellum; #ROIs 90 – excluding cerebellum). All small-world parameters were calculated at K = 10 and synchronization threshold T = 0.05.

### Global synchronization: small-worldness

In order to perform a whole-brain graph analysis, the individual networks representing every subject's synchronization patterns were computed based on their respective synchronization matrices. Corrected C (γ) and L (λ) were calculated per group as an index of the original C and L values and their randomized surrogates C-s and L-s. Importantly, the topologic descriptors are consistent as neither the removal of the cerebellar regions nor the atrophy correction using midspace as alternative registration template did affect the graph analysis results (Table 1; results per parameter are threefold, corresponding to registration to standard space with and without cerebellum and registration to midspace without cerebellum).

Mean C and L were also expressed as a function of threshold T (Figures 3a and 3b respectively). Across the range of the values of T analyzed (0.01<T<0.05) the mean clustering coefficient C (Figure 3a) and characteristic path length L (Figure 3b) of both groups slowly increase to plateau at the higher T values tested. However, L reflects clear differences between the groups: values of L are consistently lower (i.e. shorter path length) for the AD group compared with those of the control group. Compared to controls, the characteristic path length of AD functional networks is closer to the theoretical values of random networks (Table 1; AD = 1.53±0.15; controls = 1.69±0.22). The difference between AD and controls is significant (p<0.05) across a wide range of thresholds, the most significant difference found at T = 0.05 (t-test, p<0.01; Figure 3b).

The same parameters, C and L were calculated as a function of K (5<K<15). As degree K increases, the value of C increases. No significant differences were found between the groups for C for any value of K (Figure 3c). Similarly, as K increases the values of L decrease (higher mean number of edges per vertex allowing for shorter pathways between vertices). Importantly, L is consistently lower for AD, showing significant differences between AD and control groups for a wide range of K values, the highest of which is found at K = 10 (L shorter in AD patients; t-test, p<0.01; Figure 3d).

Both C and L parameters for AD and control groups are plotted together with the theoretical values for random and ordered (regular) ring-like networks (Figure 3a–d; ordered graph: C = 3/4, L = N/2K; random graph: C = K/N, L = ln(N)/ln(K)). The values of C obtained for experimental and control groups fall in between random and ordered clustering coefficient characteristic values; the characteristic minimal path lengths L for both groups are also intermediate between those for random and ordered graphs. Furthermore, the values of C and L calculated on the bases of resting state BOLD synchronization are similar to those computed based on magnetoencephalographic measurements [26].

The corrected path length (λ) did not correlate with severity of dementia, premorbid intelligence or depression scores for any of the experimental groups (partial correlation, corrected for age, gender and years of education: MMSE: AD r = −0.45; p = 0.10; OC r = 0.38; p = 0.12); NLV: AD r = −0.02; p = 0.93; OC r = −0.10; p = 0.69; GDS: AD r = −0.48; p = 0.08; OC r = 0.35; p = 0.16).

### Synchronization changes distribution

To explore in detail the origin of the different characteristic path length between AD and control groups, we performed a post-hoc analysis of the local synchronization differences between both groups. Similarly to the graph analysis results, the pattern of SL regional differences between AD and controls was found unchanged regardless of the inclusion of the cerebellum or the registration procedure. The synchronization patterns displayed in the matrices of both groups are similar in their topology (Figures 1 and 2; 116 ROIs in standard space), the differences being regional synchronization intensities. These group differences were calculated with 2-tailed t-test, p<0.05 uncorrected. Additionally, a non-parametric cluster-based permutation test was performed to control for the family-wise error rate over the 12 levels of K. We found one significant cluster containing the K-levels between 6 and 15 (p = 0.004). This result confirms the robustness of the path length difference that constitutes the main result of our study (Table 1). Additionally, it demonstrates that, in the group tested, the regional pattern of synchronization differences between ADs and controls constitutes one of the few combinations of synchronization changes that produce shorter path length in AD. The consistent results obtained with different registration procedures, together with the highly significant difference in path length make it unlikely that the differential regional pattern of synchronization between AD and controls is the produce of chance. Nonetheless, the following description of the pattern of regional synchronization differences does not report distinctions at the level of individual ROIs.

**Figure 4:**
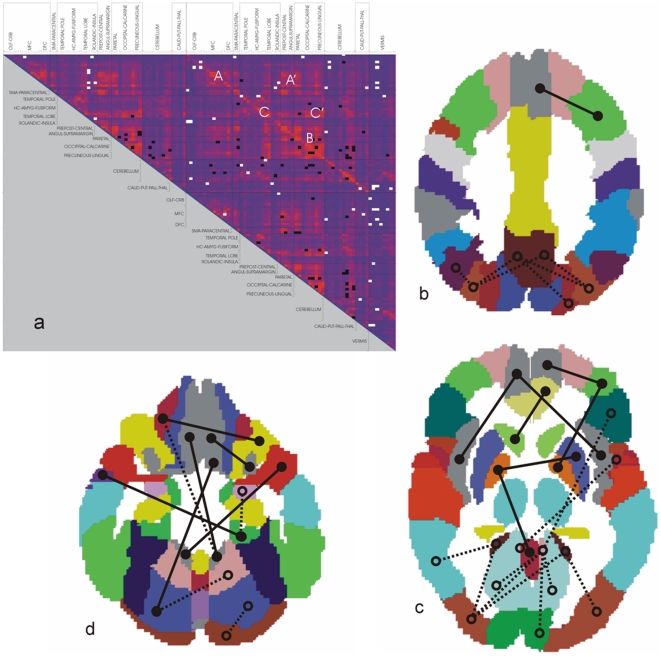
Synchronization differences. (a) Matrix of significant differences of synchronization between AD and controls (2-tail t-test, p<0.05 uncorrected). The white and black dots represent brain areas pairs with increased and decreased synchronization in AD respectively. (b-d) A subset of connectional differences corresponding to the matrix (a) are plotted at 3 superior-to-inferior levels through the AAL brain template: (b) = z53; (c) = z73; (d) = z111. Lines depict synchronization between pairs of regions: solid lines  =  enhanced synchronization; dashed lines  =  reduced synchronization. Note the pattern of generalized posterior (parietal and occipital) synchronization reductions and increased frontal synchronization.

Figure 4a represents the matrix of significant differences of synchronization levels between AD and controls. The white dots represent brain areas pairs with increased synchronization in AD while the black dots stand for reduced synchronization with respect to control subjects. Individual connectivity differences derived for the synchronization changes are represented at 3 superior-to-inferior levels through the brain shown in Figure 4 (b–d).

The majority of the relative increases in both inter- and intrahemispheric synchronization in AD belong to frontal brain regions, particularly the orbitofrontal cortices but also the medial frontal cortex. Subcortical structures including the corpus striatum (caudate nucleus, putamen and pallidum) and the thalamus also show more synchronization with the frontal cortices in the AD group, particularly with the orbitofrontal cortex (Figure 4b–c). The temporal lobes demonstrate an asymmetric pattern with increased synchronization in the right hemisphere, in contrast with decreased left synchronization. This asymmetry is maintained in its relation with the frontal lobes: only the right temporal regions show increased synchronization with the right frontal lobe (Figure 4d). Furthermore we found increased interhemispheric coupling between the temporal poles. Relative to control group, the AD group shows a widespread reduction in mean synchronization involving the caudal functional connections of the temporal lobe and, importantly, concentrated at the parietal and occipital cortices, including the precuneus. It is in the parietal and occipital cortices where the pattern of changes is clearer, with generalized synchronization decreases affecting both brain lobes (Figure 4a–c). Furthermore, the vermis in AD shows higher synchronization, particularly with the frontal cortex. Similarly, the cerebellum also shows a relative increase in synchronization with the frontal lobe and widespread synchronization decreases with the parietal and occipital cortices (Figure 4d).

**Figure 5:**
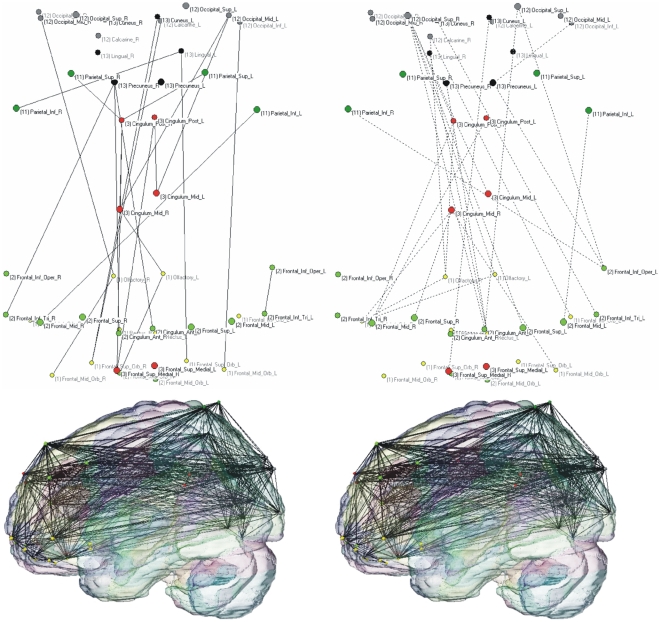
Pattern of long-distance functional connections. Connectivity pattern of AD and healthy groups (left and right panels respectively). Color-coded vertices correspond to individual AAL ROIs included in the orbital (yellow), medial (red) and dorsolateral frontal regions (light green), parietal (deep green) and occipital lobes (gray), the cuneus and lingual cortices (black). Upper 2-dimensional graphs (orientation front-down and left-right) represent the changes in functional connections: solid lines (left) correspond to increases and dashed lines decreases in connectivity in AD (right). Lower figures (orientation front-left) represent the projections of AD (right) and healthy networks (left) embedded in a 3-dimensional AAL brain template. The graphs demonstrate a net loss of long-distance fronto-parietal and fronto-occipital functional connections (for both groups, T = 0.05; K = 10).

The regional changes in synchronization described are translated into abnormal global connectivity patterns, particularly evident regarding long-distance functional connections. Figure 5 illustrates the new pattern of long distance connections in AD, demonstrating a net loss of functional connections between the frontal lobe and the parietal and occipital cortices (Figure 5a and 5b, AD and healthy connectivity patterns respectively).

In summary, both hemispheres show a similar pattern on internal synchronization differences between the experimental groups: most of the increased AD synchronization involves the frontal cortices, while generalized decreases are located at the parietal and occipital regions. Furthermore, there is a remarkable interhemispheric asymmetry as coupling of the lateral temporal lobe with the orbital, medial and dorsal frontal cortices increases within the right hemisphere. The effect is opposite in the left hemisphere, where the synchronization of the temporal lobe only shows reductions as compared to the control group. Therefore, a typical AD pattern emerges in which frontal regions of the brain show relatively higher mean levels of synchronization in contrast with reduced functional coupling affecting parietal and occipital cortices (Figure 4a for overall pattern). This effectively translates in a global reduction of functional links between frontal and caudal brain regions (Figure 5).

## Discussion

### Summary of the results

This study demonstrates a graph analysis of resting-state fMRI BOLD time-series of Alzheimer patients and healthy controls using synchronization likelihood (SL) as a measure of functional coupling. The structure of the whole-brain average functional network in the control group is of a small-world network. With equivalent global average synchronization, the network in AD is distinguished to the controls' network by a relative randomization of its architecture driven by a different connectivity pattern.

### Graph analysis and Small-world classification

The analysis of whole-brain functional connectivity poses particular challenges. Arguably the most complex object in the universe, the human brain generates an extensive and intricate map of functional relationships. Due to the vast number of functional relationships possible, the examination of its detail structure is often selective, focusing on isolating subnetworks from the global network. We have demonstrated that individually defined functional subnetworks [32] can also be interpreted as constituents of a global activity pattern. The application of graph analysis to a whole-brain functional network reflects the aggregated functional topology of the brain networks in a few measures with clear theoretical meaning. The global functional topology can then be expressed in essential organizational principles, like clustering and connectedness [28], [49], [50], [51], [52], [53], [54] and be used to interpret global functional dynamics within a network model [18], [23]. Although the small-world network architecture is only an approximate model of the brain networks, i.e. it doesn't account for physical distances between network elements, it provides a high-level description of the implications of AD-induced changes in the global functional state of the AD brain.

### Small-world model: increased connectedness and randomization in AD

The discrete connectivity changes induced by AD result in a global reduction of the network's characteristic path length. Our results are confirmed in a recent study by Stam et al., which reexamines functional connectivity in AD by means of magnetoencephalograpy [35]. Similar to our methodology, Stam et al. include the calculation of L using the harmonic instead of arithmetic mean, to better handle infinite path lengths between disconnected edges. Furthermore, it addresses the issue of volume conduction using a new measure of functional synchrony (phase lag index), comparable to SL but less influenced by common sources. This refined analysis results in a reduced path length in AD that is in contrast with earlier reports [18], [27]. Therefore, connectivity characterizing low-frequency synchronized brain activity in AD, measured both by MEG and fMRI, seems to move away from the optimal small-world network configuration.

The same trend towards randomness in AD networks described above has been reported by Supekar et al. [17]. In contrast with our results, Supekar et al. found a reduced clustering coefficient in AD patients but an unchanged path length. Importantly, the basic regional changes in functional correlations detailed by Supekar et al. extensively coincide with the changes we describe using synchronization likelihood: (1) AD increased functional connectivity within the frontal cortices and between them and corpus striatum and thalamus, and (2) decreased between the temporal lobe and parietal and occipital cortices. The age of the participants could be a confounding factor in the Supekar et al. study as the population described included a wide age-range (+/−18.5 years approximately) compared to the one we have studied (+/−6.5 years). Considering however the high level of coincidence between Supekar et al. functional correlations and the synchronization patterns hereby presented, only methodological differences could explain the disparity of the graph analysis results. An important distinction between both studies is the application of spatial smoothing by Supekar et al. (4 mm full width half maximum Gaussian kernel). This preprocessing step implies local time-series averaging, which can generate spurious synchronization between neighboring voxels. The effect of local data smoothing can introduce differences between both groups examined due to the higher degree of cortical atrophy characteristic in AD, which emphasizes partial volume effects resulting in a lower cluster coefficient for this group. In order to prevent this problem, *in the present study time-series averaging was only applied within the anatomic regions defined by the AAL brain template*.

According to the principles of the small-world theoretical model, the decreased normalized path length of the AD network represents a relative randomization of the functional topology of a healthy brain network. The establishment of new intercluster connections could explain the decreased path length. In AD, new intercluster abnormal connections convey the risk of generating an uncontrolled flow of information through the entire network [50], [51].

### Frontal-coupling and caudal-decoupling: long distance connectivity loss

Most of the synchronization increases are localized in the frontal lobes including the orbitofrontal, medial and dorsal frontal cortices. Parietal, occipital cortices and precuneus harbor most of the synchronization reductions. This regional distribution of synchronization changes confirms the reported lack of functional connectivity in subjects with amnesic mild cognitive impairment between the temporal lobe structures and posterior brain areas [55] and it is compatible with the suggested anterior-posterior disconnection in AD [9], [56], [57]. Using synchronization likelihood as a measure of functional connectivity in magnetoencephalographic recordings, Stam et al. [27] reported similar fronto-parietal disconnections in AD in both alpha and beta frequency bands. The present data set was analyzed with tensor PICA, also demonstrating a decrease in activity in AD patients compared with healthy subjects within the dorsal visual-spatial attention system and DMN [7], [8], [55], [58], [59], [60]. Our result also confirms previous reports showing parietal deactivation as one of the characteristic features of the Alzheimer's brain activity patterns [61], [62], [63] for a review].

The parietal decrease in synchronicity coincides with the AD network topology, reflecting an overall decrease in the number of direct connections between frontal lobes and the parietal and occipital cortices. The loss of long distance connections supports the view of AD as a disconnection syndrome [1].

The pattern of increased dorsolateral prefrontal and cingulate cortices activity in AD had been reported in the context of goal-directed tasks [14], [56]. Correlated to improved memory performance in AD patients, prefrontal activity has been suggested to facilitate a compensatory function [14]. Our data demonstrate that these abnormal increases of functional synchronization within the frontal cortices are also present during resting-state, confirming an earlier report [9]. Moreover, this AD frontal activity pattern is clearly distinct from the hypofrontality syndrome characteristic of schizophrenia [34].

### Increased fronto-temporal synchronization

Within the DMN, the combination of individual ICA and template matching procedure for group analysis [8] demonstrated local bilateral decreases of activity in the hippocampus. This result was not replicated by applying tensor PICA, although an ulterior application of the template matching procedure on the same data set did render similar results [11]. However, such a reduction in activity must be interpreted as relative to the other the components of the DMN, obviating the functional relationships of the hippocampus and the temporal lobe with non-DMN areas. In contrast with the studies above, our findings point to an asymmetric connectivity pattern, with increased synchronization in the right hemisphere, in contrast with decreased left side synchronization both locally and with the frontal lobes. Importantly we found increased interhemispheric coupling between the temporal poles. The observation of a global asymmetric pattern of changes between rostral and caudal temporal lobe connectivity in Alzheimer disease could explain the lack of consistency in previous studies. In agreement with the described functional asymmetry in the temporal lobe, Karas et al. also found asymmetry in gray matter loss using VBM analysis, reporting greater left than right mediotemporal lobe atrophy in AD [64].

### Functional connectivity networks compared to cortical thickness networks in AD

**Figure 6:**
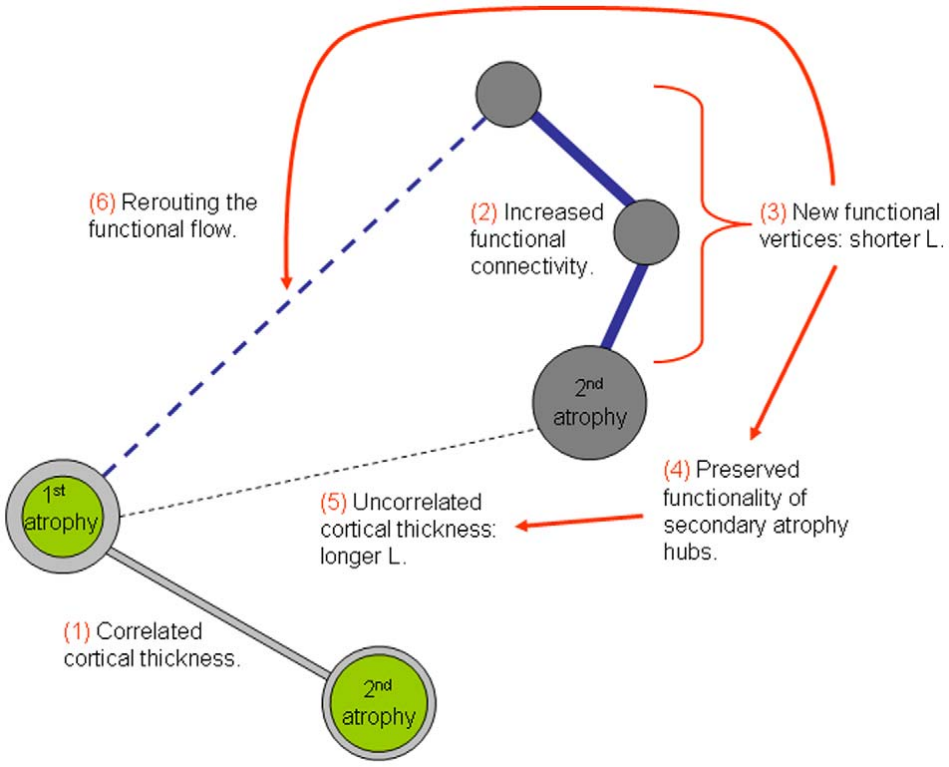
Effect of increased functional connectivity in secondary atrophy sites. Theoretical interpretation of simultaneous longer atrophy and shorter functional path length in AD. Initial correlated atrophy between vertices (1) is decoupled due to compensatory functional connectivity involving secondary atrophy vertices (2). The appearance of new functional edges (3 - shorter functional L) results in preserved functionality of secondary atrophy vertices (4) and the disappearance of cortical thickness edges linking to the primary atrophy vertex (5 - longer structural L). The rerouted functional flow could still gain partial access to the primary atrophy site and the rest of the network (6).

The integration of structural and functional networks represents a critical challenge in connectivity research. The elegant study by He et al. [19] on the analysis of cortical thickness intercorrelations exemplifies this difficulty, describing a structural network characterized by a long path length that is in direct contrast with the presently described shorter functional path length. How is it possible to imagine the structure of a network with lower structural connectivity and yet supporting higher functional connectivity? The effect of compensatory activity on secondary atrophy sites can offer a tentative explanation. Localized cortical atrophy might result from secondary axonal and trans-synaptic degeneration following primary injury ([65]; [66] for a review). This in turn can induce changes in metabolism at brain regions far removed from the primary atrophy site [67], some of which are of compensatory nature [14]. We hypothesize that, in Alzheimer's disease, compensatory increases in functional connectivity could help maintain the functionality and structural integrity of secondary atrophy sites, with two main consequences at network level: 1) relative increases of cortical thickness path length, and 2) relative decreases of functional path length (Figure 6). Additionally, the rerouted functional flow could still gain partial access to the rest of the network.

### L reduction in AD: inter- and intra-cluster connectivity changes

According to the small-world network model proposed by Watts and Strogatz [23], a reduction of the small-world parameter L depends on connectivity decreases within functional clusters and their replacement by inter-cluster connections. An interaction of both mechanisms explains the shorter L in AD. The identification of the vertices of the network and the classification of both functional and structural changes they undergo in AD could describe the balance responsible for the decrease in path length [25], [59]. The effect of lesion modeling in network robustness reveals the importance of this objective as the influence of the lesion is likely to depend on the global embedding of the affected vertex. In the context of structural networks, the removal of inter-cluster vertices generates network-wide effects, while the influence of intra-cluster vertices remains within the cluster [68]. In particular, AD structural networks have been reported to be more vulnerable to attacks on vertices with higher connectedness [19]. When lesions are modeled on functional networks, Stam et al. show that network changes in AD are driven mainly by a reduction of connections between highly connected vertices [35]. It is tempting to speculate that, as well as with lesion modeling, the effect of compensatory activity hypothesized earlier (Figure 6) might depend on the type of vertex implicated.

Although local processing advantages can be drawn from a reduced L, the overall randomization of the network represents a less optimal network organization [30], [31], [69]. The examination of the system in patients in transition phases to AD, like MCI, will help elucidate whether the described frontal hyperconnectivity is part of the compensatory mechanisms described in early Alzheimer patients [14].

### Graph analysis in Alzheimer: methodological considerations

Atrophy is a major factor in Alzheimer disease, and its brain distribution and correlation to cognitive decline are well described [64], [70], [71]. Due to local atrophy, the local average of time-series per ROI could include signal derived from voxels corresponding to CSF within the region of interest defined by the original AAL mask, thereby introducing artificial differences between AD and control subjects' SL values for the same regions. We controlled for artificial differences in functional coupling related to differential atrophy rates by calculating a custom brain or midspace. Once registered to the midspace, the resulting individual functional sequences approximate the average size and shape of both experimental groups, limiting atrophy and registration-related artifacts. We hypothesized that, in the case of significant differences in brain size due to atrophy, the graph analysis results would vary depending on the registration to the “control-based” MNI152 or the “AD-weighted” midspace templates. The different transformation procedures alter the original data resulting in slightly different SL values. Importantly, both global synchronization and the graph descriptors calculated remain unchanged, independently of the registration method. This approach constitutes a conservative correction for atrophy in order to avoid potential overcorrection affecting small-sized ROIS, which would lead to an artificially high signal.

Different SL values between subjects could also influence the construction of the graphs. To study the differences in actual topology between the AD and control graphs constructed, the graph parameters C and L are computed per subject at a given average number of edges per vertex (K), implying the application of different thresholds to different subjects. Although there are no significant differences for SL average values between the groups, the fixing for K means that edges computed for different graphs do no longer all have the same meaning as those in patients can be based upon weaker synchronization levels. Nevertheless, controlling for K is the most rigorous way to compare networks in terms of ‘pure’ topology [18], [20].

Graph analysis provides a way to handle complex data sets with numerous pair-wise comparisons in a relatively simple way, reducing the number of comparisons to a small number (normalized clustering coefficient and path length, for instance). Based on the group differences demonstrated with graph analysis, a post-hoc analysis was performed at the level of regional synchronization differences between the groups with significantly different path length. Similar regional patterns of significant synchronization differences are generated regardless the inclusion of the cerebellum (defined as 26 subregions) and the different registration methods applied (standard space or custom midspace). The consistent results of the regional synchronization level post-hoc analyses provide additional confidence for the interpretation of the synchronization changes discussed. However, given the high number of tests performed, local differences of synchronization level comparisons cannot be analyzed with confidence. Classic solutions for the multiple comparisons problem (like Bonferroni correction or FDR) are too rigorous, and clustering-based corrections (grouping of connected individually significant voxels by imposing a single cluster extent threshold) may not be appropriate given the different size of the original ROIs (see Figure 4). Instead, we performed a cluster-based permutation test, randomly repartitioning AD patients and controls over two groups. By modeling data sets representing all possible combinations of patients and controls, we tested for the likeliness of our original result. The cluster-based permutation test corroborates the high significance of the original path length differences (p = 0.004). This result further supports the original pattern of synchronization changes as the combination most likely to generate the AD-related path length difference in our sample of the population. Nevertheless, we conservatively describe only patterns at a regional level and not pair-wise ROI differences.

### Summary

In summary, we have demonstrated AD-induced changes in global brain functional connectivity by analyzing low frequency BOLD fMRI fluctuations during resting state condition. Critically, this study demonstrates a randomization of the brain functional networks in AD, suggesting a loss of global information integration in disease. Our results describe the functional differences between frontal and the parietal and occipital lobes in AD, supporting the anterior-posterior disconnection theory.

## Supporting Information

File S1 Mathematical background of dynamical systems theory, generalized synchronization and synchronization likelihood.
(0.03 MB DOC)
