# Supplementary material for: Loss of ‘Small-World’ Networks in Alzheimer's Disease: Graph Analysis of fMRI Resting-State Functional Connectivity
Source: PLoS One. 2010 Nov 1;5(11):e13788. doi: 10.1371/journal.pone.0013788 (PMC2967467; doi:10.1371/journal.pone.0013788)
Supplement: File S1 — Mathematical background of dynamical systems theory, generalized synchronization and synchronization likelihood. (0.03 MB DOC) [file pone.0013788.s001.doc]

**Supporting Information S1. Mathematical background of dynamical systems theory, generalized synchronization and synchronization likelihood**

**Here we briefly recapture some basic notions from dynamical systems theory and give a formal definition of synchronization likelihood. The key step is to reconstruct, from a time series of observations, the *attractor* of the underlying dynamical system.**

**Assume we have two time series x_i_ and y_i_, where the index *i,* i = (1..N), denotes discrete time. From each of these time series we construct a series of *m* dimensional vectors X_i_ and Y_i_ in state space with the method of time-delay embedding (Takens, 1981) as follows:**

$X_{i}=(x_{i},x_{i+L},x_{i+2\times L},x_{i+3\times L}...,x_{i+(m-1)\times L})$ **[1]**

**where *L* is the time lag, and *m* the embedding dimension (m << N). From a time series of N samples, N-(m × L) vectors can be reconstructed. Takens has proven mathematically that, for a sufficiently high *m*, the reconstructed vectors correspond to the attractor of the underlying dynamical system (Takens 1981). The attractor is a geometric object in the state space (or phase space) of a dynamical system, which represents its fundamental characteristics such as its degrees of freedom, sensitive dependence on initial conditions and conservative or dissipative dynamics. The general equation for a dynamical system is:**

$\frac{dX}{dt}=G(X,\mu,\varepsilon)$ **[2]**

**Here μ represents a set of parameters and ε is noise. A dynamical system X is said to be linear if G is linear (system of linear differential equations), otherwise X is non linear. The attractor of the dynamical system is the geometric repesentation of the solution of the equations of motion. Interactions between two dynamical systems X and Y can be described by the concept of *generalized synchronization*, as introduced by Rulkov et al. (1994):**

$Y=F(X)$ **[3]**

**This equation says that the state of dynamical system Y (the response system) is a function F of the state of dynamical system X (the driver system). The function F maps each point of X on a corresponding point of Y. The only requirement is that F is locally smooth. The coupling between X and Y is said to be linear when F is linear, and non linear if F is non linear.**

**The synchronization likelihood is an algorithm to determine the strength of generalized synchronization between two systems X and Y, where X and Y are obtained from time series by time delay embedding [1]. Since the method is based upon what can be considered a local linear approximation of F, it can handle linear and well as non linear cases of F. The fundamental idea is that two points on X that are very close together should be mapped to two points on Y that are also very close together if [3] holds.**

**Synchronization likelihood is defined as the conditional likelihood that the distance between Y_i_ and Y_j_ will be smaller than a cutoff distance r_y_, given that the distance between X_i_ and X_j_ is smaller than a cutoff distance r_x_. In the case of maximal synchronization this likelihood is 1; in the case of independent systems, it is a small, but nonzero number, namely P_ref_. This small number is the likelihood that two randomly chosen vectors Y (or X) will be closer than the cut-off distance r. In practice, the cut-off distance is chosen such that the likelihood of random vectors being close is fixed at P_ref_, which is chosen the same for X and for Y. To understand how P_ref_ is used to fix r_x_ and r_y_ we first consider the correlation integral:**

$C_{r}=\frac{2}{N(N-w)}\sum_{i=1}^{N} \sum_{j=i+w}^{N-w} \theta(r-\left|X_{i}-X_{j}\right|)$ **[4]**

**Here the correlation integral C_r_ is the likelihood that two randomly chosen vectors X will be closer than r. The vertical bars represent the euclidican distance between the vectors. N is the number of vectors, w is the Theiler correction for autocorrelation (Theiler, 1986) and θ is the Heaviside function: θ(X) = 0 if X >=0 and θ(X) =1 if X < 0. Now, r_x_ is chosen such that Cr_x_ = P_ref_ and r_y_ is chosen such that Cr_y_ = P_ref_. The synchronization likelihood between X and Y can now be formally defined as:**

$SL=\frac{2}{N(N-w)p_{ref}}\sum_{i=1}^{N} \sum_{j=i+w}^{N-w} \theta(r_{x}-\left|X_{i}-X_{j}\right|)\theta(r_{y}-\left|Y_{i}-Y_{j}\right|)$ **[5]**

**SL is a symmetric measure of the strength of synchronization between X and Y (SL_XY_ = SL_YX_).In equation [5] the averaging is done over all i and j; by doing the avering only over j SL can be computer as a function of time i. From [5] it can be seen that in the case of complete synchronization SL =1; in the case of complete indepence SL = P_ref_. In the case of intermediate levels of synchronization P_ref_ < SL < 1.**

**In the present study the parameters were set as follows: w1 = 1, lag *L* = 1; embedding dimension *m* = 6; P_ref_ = 0.05. We computed for each of the 116 or 90 time series (depending of the number of ROIs used, with or without cerebellum) the synchronization likelihood between a particular time series corresponding to a single ROI and all other.**
